# Tuberculous Peritonitis Treated without Operation

**Published:** 1909-08-14

**Authors:** 


### TUBERCULOUS PERITONITIS TREATED WITHOUT OPERATION.

The first successful operation for tuberculous peritonitis was an accident, for laparotomy was being performed under the idea that the patient was suffering from an ovarian cyst. The case was one of the ascitic type, and when recovery resulted the cure was not unnaturally attributed to the beneficial effect of the laparotomy. This was in the days when it was less clearly realised than it is now that any tuberculous lesion may recover completely under careful advice as to the mode of living and as to the food to eat, and since then our opinions have been considerably modified. Careful hygienic treatment in tuberculous cases has come to be regarded as of the utmost value at least as a preliminary to more radical lines of interference, and the limits of usefulness of operative treatment have been much restricted.

Nevertheless this original case of recovery after laparotomy for tuberculous peritonitis rapidly led the younger school of medical men to suppose that the treatment of all tuberculous peritonitis —or if not of all tuberculous peritonitis, at any rate of that form in which ascites is a prominent feature —is essentially operative. It was not long before those who had access to large numbers of cases came to the conclusion that laparotomy does not do for them anything like the good that is expected.

### A Difference of Opinion.

Indeed, both clinical and experimental results show that operative measures in the earlier stages of the disease are positively harmful. Even when all would be agreed that the case before them was one in which an operation should be done, some would advocate simple incision through the abdominal wall and re-suture; others, incision and the letting out of the fluid before re-suture; others, in addition to letting out the fluid, disinfection; some counsel the breaking down of adhesions, others deprecate this; some advise that sunlight should be let in to the peritoneal cavity; there is no unanimity of opinion as to what should be done, so that many have argued that it does not matter what is done so long as simple laparotomy is performed. Others, on the other hand, argue that the cases should for the most part not be operated upon at all.

### The Case for Non-Operative Treatment.

The results of an investigation by Dr. Stone, of the Massachusetts General Hospital, may help us in forming some opinion upon these questions. He traced the subsequent histories of 122 cases, some operated upon and some not, and definitely concluded that operation is not to be advised early, nor indeed until the effects of other treatment have been carefully watched first. He finds that several patients with well-marked ascites begin to improve almost suddenly, and he points out that had these cases been operated upon it is more than probable that the rapid improvement would have been attributed to the operation. If Gelpke's view that the peritoneal exudate contains bactericidal substances is true it would seem to be decidedly wiser to leave it in the body than to remove it.

### Some Noteworthy Conclusions.

The general conclusions that can be drawn from Dr. Stone's long paper are as follows:—

First, that the outset and course of the disease are so extremely variable that it must always be a matter of great difficulty to determine whether recovery or the reverse are due to any particular line of treatment that has been followed. There are patients in whom the symptoms are lit up in a sudden and stormy manner, both in ascitic and in non-ascitic cases. On the other hand, the tuberculous peritonitis may seem to have been cured, or even to have been non-existent, and yet it is merely latent so far as symptoms are concerned; it can remain so for months or years and yet light up again, just as in the case of pulmonary phthisis.

Secondly, the source of infection is unknown; it is presumed that the peritoneum becomes infected from the intestine, and therefore from the food—milk, for instance; but this is very difficult to prove; it is noteworthy, however, that Dr. Stone finds none of his cases to have arisen from tuberculous disease of the Fallopian tubes, as some authors think not uncommon, nor from similar disease of the vermiform appendix.

Thirdly, although it has sometimes been stated that tuberculous peritonitis is commoner in girls than in boys—as it would require to be if tuberculous disease of the uterine appendages were its common precursor—Dr. Stone finds that males and females are affected in exactly equal proportions.

Fourthly, it is possible for the ascitic fluid to begin to clear up almost suddenly in cases that have remained stationary for weeks.

### A Point as to Treatment.

Fifthly, that in the cases with ascites the best treatment is to keep the patient at rest in bed or upon a couch with the fresh air and good food régime that would be adopted in a corresponding case of pulmonary phthisis. Operation should not be advised, at any rate until this course has been followed for at least six or eight weeks, and even then an operation should only be undertaken when there is some distinct indication for it, such as distress from the abdominal distension. In any case, whether laparotomy is performed or not, the mode of life and the dietary need the same careful attention after the arrest of the disease as before it if recurrence is to be prevented.

